# Controlling Much? Viral Control of Host Chromatin Dynamics

**DOI:** 10.1146/annurev-virology-100422-011616

**Published:** 2024-08-30

**Authors:** Laurel E. Kelnhofer-Millevolte, Edward A. Arnold, Daniel H. Nguyen, Daphne C. Avgousti

**Affiliations:** 1 Department of Molecular and Cellular Biology, University of Washington, Seattle, Washington, USA; 2 Medical Scientist Training Program, University of Washington, Seattle, Washington, USA; 3 Department of Microbiology, University of Washington, Seattle, Washington, USA; 4 Human Biology Division, Fred Hutchinson Cancer Center, Seattle, Washington, USA

**Keywords:** chromatin, histones, herpesvirus, adenovirus, latency, nucleus

## Abstract

Viruses are exemplary molecular biologists and have been integral to scientific discovery for generations. It is therefore no surprise that nuclear replicating viruses have evolved to systematically take over host cell function through astoundingly specific nuclear and chromatin hijacking. In this review, we focus on nuclear replicating DNA viruses—herpesviruses and adenoviruses—as key examples of viral invasion in the nucleus. We concentrate on critical features of nuclear architecture, such as chromatin and the nucleolus, to illustrate the complexity of the virus-host battle for resources in the nucleus. We conclude with a discussion of the technological advances that have enabled the discoveries we describe and upcoming steps in this burgeoning field.

## 1. CHROMATIN ORGANIZATION IN THE NUCLEUS

Chromatin, which is a combination of DNA, RNA, and proteins, specifically refers to the state of host DNA prior to condensing during prophase into the more widely recognized chromosome. Host chromatin is organized into units called nucleosomes that consist of approximately 147 bp of DNA wrapped around an octamer of histone proteins (1). Post-translational modifications on these histone proteins, including methylation and acetylation, are integral to regulation of gene expression and have been extensively reviewed (2). Regions of chromatin that are transcriptionally active are broadly classified as euchromatin, while regions that are transcriptionally repressed are called heterochromatin (2, 3). Some regions of heterochromatin, such as telomeres and centromeres, are maintained in a permanently inactive state and referred to as constitutive heterochromatin (4). Conversely, other regions of heterochromatin, termed facultative heterochromatin, are dynamically expressed at different times and are associated with histone modifications such as trimethylation on the lysine 27 residue of histone H3 (H3K27me3), H3K9me3, and the histone variant macroH2A (5, 6) (see sidebar titled Abbreviations List). These histone modifications (or marks) are critical for defining the local conformation of chromatin, thereby dictating access to genes for expression or repression as needed by the cell. For example, once a cell has differentiated and expression of developmental genes is no longer needed, these genes must be repressed to support cell identity. Conversely, immune signaling genes are expressed in response to stimulus and must be poised for rapid induction to defend the cell when needed. The combination of histone marks on a gene locus can also have important outcomes on how that gene may be expressed, often referred to as a histone code or cross talk language (7, 8). As chromatin marks come together and influence local and global compaction, the 3D nuclear landscape takes shape.

Advances in sequencing and chromatin capture technologies have brought the importance of global chromatin architecture to the forefront of molecular biology (Table 1). These techniques have aided the discovery of complex folding of the chromatin in a highly specific and well-regulated manner. Chromatin folding is often reported as looping (9, 10) and enables regions of DNA that are several kilobases away in primary sequence to interact in three dimensions. Uncovering this layer of complexity has revealed that enhancers, promoters, and other regulatory elements may have overlapping functions (11). Further, studying 3D interactions within the genome showed that transcriptional activation may not occur as isolated events but rather in hubs or frequently interacting regions (FIREs) that consolidate resources to specific physical locations for greater efficiency (12). Larger regions of interaction are referred to as topologically associating domains (TADs) (13), spanning more than 800 kb on average with around 2,200 TADs per genome and multiple combinations of histone marks per TAD (14). TADs are further organized into active A compartments or more silent B compartments, which in turn make up chromosome territories (15). In addition to compartmentalizing gene regulation, these larger areas also physically support the structure of the nucleus (16).

Because viral infection requires host cell resources, this necessitates control of the host genome and the host chromatin. As such, viral infection leads to disruption of normal chromatin function at many levels, from hijacking histone modifying enzymes to exploiting global changes in nuclear morphology. One key protein involved in the organization of chromatin that is discussed in this review as it pertains to viral infection is the CCCTC-binding factor, CTCF. CTCF is best known as a transcription factor but has multiple roles as a repressor and insulator and defines the boundaries between chromatin domains or TADs (17). The structural role of CTCF is largely due to its interaction with the cohesin protein complex (15), a ring complex that recognizes CTCF and helps stabilize loops and TADs (18). CTCF action brings enhancer regions into close physical proximity with promotors to drive transcription, though it can also insulate genes from enhancers to repress transcription. Because of this critical role in TAD maintenance and gene expression, many viruses exploit CTCF and chromatin looping to regulate their own gene expression.

**Chromatin:**

the state of host DNA, associated proteins, and RNA when the cell is not dividing

**Histone:**

small basic proteins around which DNA wraps to form nucleosomes; the nucleosome is made up of an octamer of core histone proteins H2A, H2B, H3, and H4

**Heterochromatin:**

originally defined as dense staining by electron microscopy and refers to transcriptionally inactive chromatin

**H3K27me3:**

denotes the post-translational modification of trimethylation on histone H3 at lysine 27 and is generally associated with transcriptional repression

**H3K9me3:**

denotes the post-translational modification of trimethylation on histone H3 at lysine 9 and is generally associated with transcriptional repression

**Histone variant:**

histone proteins, such as macroH2A and H3.3, encoded by separate genes that can replace core histones in the nucleosome and alter chromatin structure and function

**Chromatin capture technologies:**

sequencing-based assays used to investigate 3D organization of chromatin; includes 3C, 4C, Hi-C, and ChiaPET, among others

**Topologically associating domains:**

regions spanning hundreds of kilobases of linear genome that are spatially organized in close proximity

Surrounding the chromatin and supporting the nuclear membrane is a meshwork of proteins collectively called the lamina. The lamina is composed of two types of lamin: splice isoforms Lamin Aor C, and Lamin B encoded by a separate gene. Interlaced Lamin A/C with Lamin B supports the nuclear membrane and interacts with additional proteins such as Emerin, SUN1, and BAF (19). These components are part of the linker of nucleoskeleton and cytoskeleton (LINC) complex that anchors the chromatin to the nuclear envelope and reinforces the structure (20). When the nucleus undergoes mitosis, the lamina is phosphorylated and rearranged to facilitate division (21). Mutation of these key proteins can cause disruption to the lamina structure and lead to diseases known as laminopathies (22). Enveloped nuclear replicating viruses such as herpesviruses are too large to enter or exit through the nuclear pores. Thus, the nuclear lamina presents a barrier to nuclear virus infection. To overcome this block, viruses have evolved mechanisms to disrupt the lamina to facilitate infection, recently reviewed in depth (23).

Within the nucleus also resides the well-defined nucleolus where ribosomal RNA (rRNA) is transcribed and ribosomes are assembled. It is currently thought that the nucleolus exists as a phase-separated subcompartment of the nucleus that houses RNA and proteins mainly associated with ribosomal biogenesis (24). Nucleolar morphology is important for cellular health as increased nucleolar volume is associated with cancers (25), whereas decreased nucleolar volume was observed in neurons with neurodegenerative disease (26). Studies focused on nucleolar function have defined nucleolar involvement in spindle formation, nuclear structure, cell cycle control, and stress sensing (27–29), underscoring the importance of this compartment in nuclear integrity. As viral infection takes over the nucleus, the nucleolus becomes a critical player both in sequestering proteins and as a powerhouse for the assembly of viral progeny.

## 2. HERPES SIMPLEX VIRUS REORGANIZATION OF THE NUCLEUS

Herpes simplex virus-1 (HSV-1) and herpes simplex virus-2 (HSV-2) are closely related alpha-herpesviruses that are estimated to infect up to two-thirds of the world’s population (30). Like all herpesviruses, HSV has lytic and latent phases, allowing the virus to persist through the lifetime of the host (31). Once infection is established, HSV-1 and HSV-2 maintain latency in the trigeminal and sacral ganglia, respectively, reactivating occasionally when stress triggers activation of lytic gene promoters (31, 32). Initial infection and reactivation in HSV-1 usually present as oral sores, whereas HSV-2 is more frequently associated with genital sores in healthy individuals. As population demographics shift, HSV-1 is becoming more represented as a sexually transmitted disease (33). Typically, infection and reactivation of HSV are painful for individuals, though mortality is limited. In contrast, severe complications can occur in immunocompromised individuals and neonates (34). Acyclovir or acyclovir-derived drugs that inhibit viral replication as a nucleoside analog are the primary treatment used for HSV infection (35). Acyclovir can reduce viral symptoms, but there is still no curative therapy for HSV. HSV-1 is more commonly used as a model system for herpesviruses. Due to its close homology, HSV-2 is likely to share similar biology. In this review, we refer to HSV-1 and HSV-2 collectively as HSV.

**Figure 1:**
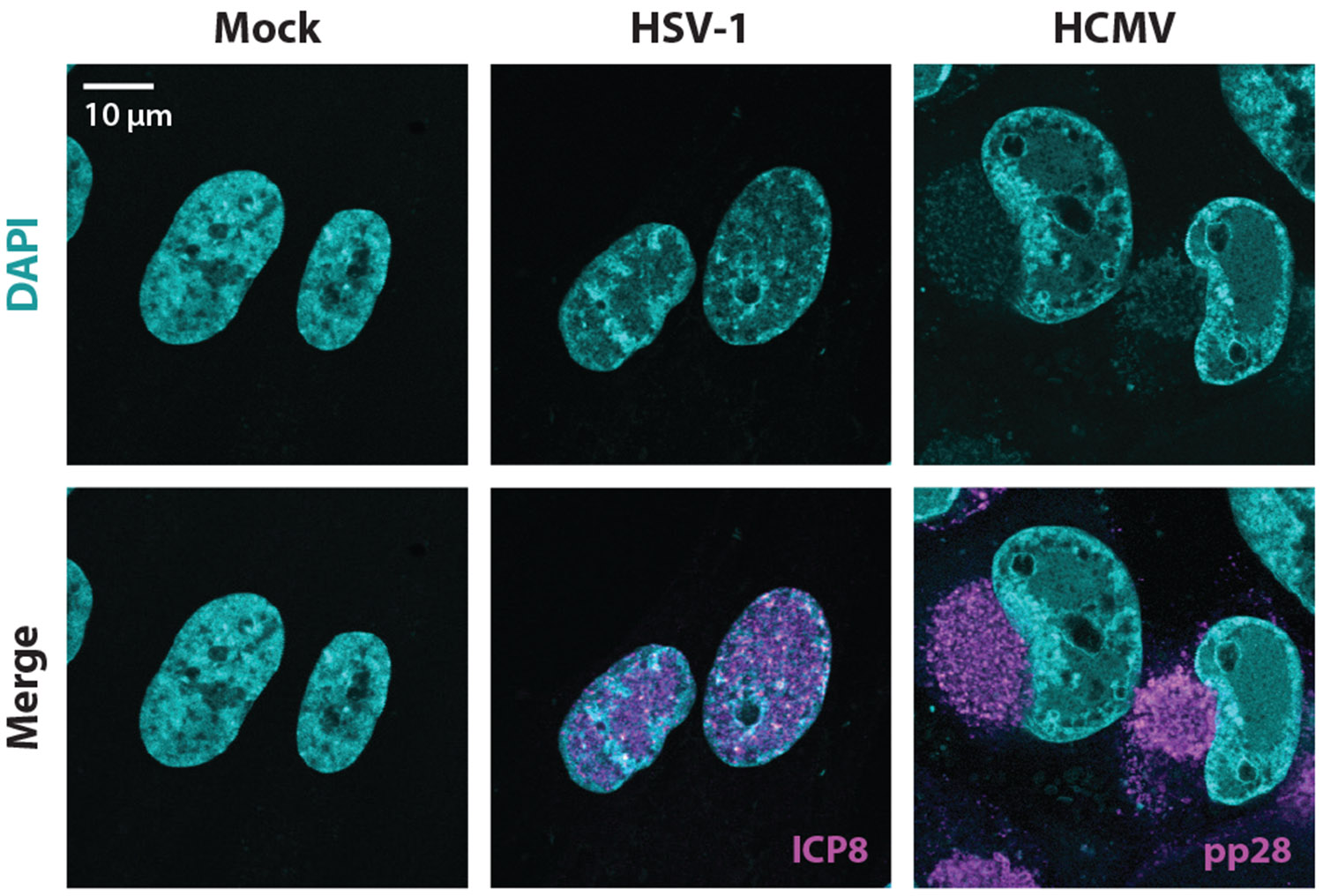
Herpesvirus infection leads to host chromatin disruption. Immunofluorescence images demonstrate lytic infections of HSV-1 (*syn17*+, 10 h post infection) and CMV (Towne, 3 days post infection) in primary HFF cells. DAPI is shown in cyan; viral proteins (ICP8 for HSV-1 and pp28 for CMV) are shown in magenta. Abbreviations: CMV, cytomegalovirus; DAPI, 4′,6-diamidino-2-phenylindole; HCMV, human cytomegalovirus; HFF, human foreskin fibroblast; HSV, herpes simplex virus.

For over a half-century, biologists have used HSV-1 as a model system for herpesviruses and for nuclear disruption. Early electron microscopy (EM) work described the most notable features of infection as the disruption of the nucleolus and “a progressive reduction of chromatin” (36, p. 368), and these large-scale disruptions were consistent across cell types (37–39). Because DNA was not actually lost, it was concluded that chromatin was redistributed rather than reduced. In addition to nucleolus loss and chromatin marginalization, the host nucleus dramatically increases in size over the course of infection (40–42) (Figure 1). In 2000, Monier et al. (40) generated 3D imaging by combining EM and confocal microscopy to demonstrate this increase. Simpson-Holley et al. (42) confirmed this finding and went further to identify host and viral factors involved, notably showing that the increase in nuclear volume is dependent on nuclear G-actin. Inhibiting G-actin prevented nuclear expansion, whereas inhibiting the cytosolic F-actin did not affect nuclear expansion during infection. These early observations built a solid foundation for more recent technological advances in biophysical techniques such as atomic force microscopy and 3D imaging to be applied to investigating changes in the nucleus induced by HSV.

**CTCF:**

CCCTC-binding factor; a transcription factor that is critical for maintaining 3D chromatin structure

**Cohesin:**

a ring-like protein complex that interacts with CTCF to stabilize chromatin loops

**Nucleolus:**

a subnuclear structure where rRNA is transcribed and ribosomes are assembled

### 2.1. Chromatin as a Barrier to Herpes Simplex Virus Infection

As soon as the HSV-1 genome enters the nucleus, the host chromatin begins to respond. In fact, injection of the bare HSV-1 genome into an isolated nucleus causes the immediate stiffening of chromatin around the nuclear periphery, measured by atomic force microscopy (43). As infection progresses and replication begins, host chromatin becomes marginalized to the nuclear periphery (40, 42). While visualization implies the physical movement of chromatin, recent work suggests that new heterochromatin is formed in the nuclear periphery by the repression of hundreds of housekeeping genes (44) (see Section 2.1.1). Because viral replication centers (VRCs) continue to expand, it is thought that nuclear expansion is likely due to a decrease in overall space. Counterintuitively, small spaces on the order of a few hundred nanometers between chromosome territories, termed corrals, increase in size during infection (45). Progeny capsids diffuse through these corrals to reach the inner nuclear membrane (INM) and begin egress (46). The movement of capsids through regions co-staining with chromatin is significantly slower than movement through regions without chromatin, indicating that host chromatin is a potential barrier to nuclear egress.

Egress from the nuclear compartment is facilitated by the nuclear egress complex (NEC), composed of viral UL31 and UL34, and supported by viral kinase Us3 (47–49). Once phosphorylation of lamin B1 by viral Us3 and host protein kinase C causes depolymerization of the lamina, capsids dock at the INM and bud into the perinuclear space (50, 51). Just before reaching the INM, progeny HSV-1 capsids in the nuclear periphery associate with regions of less dense chromatin, or channels (52). Densely stained chromatin surrounds these channels, suggesting that host heterochromatin is important for their formation. In uninfected cells, heterochromatin markers macroH2A1 and H3K27me3 at the nuclear periphery support nuclear integrity (53, 54). Depletion of these markers causes progeny capsids to diffuse more slowly and accumulate in the host nucleus, resulting in lower viral titers (44). Thus, host chromatin acts as a physical barrier to HSV-1 egress in multiple ways.

**Viral replication center:**

a virus-generated microenvironment in which viral replication occurs and viral capsids are formed and packaged

**Egress:**

the process by which progeny viral particles leave the nucleus or host cell

**Nuclear egress complex:**

conserved herpesviral proteins that facilitate the exit of progeny virions out of the nucleus; composed of UL31/UL34 in HSV and UL50/UL53 in CMV

#### 2.1.1. Host chromatin response to lytic herpes simplex virus infection.

Chromatin dynamics on the host genome regulate the transcription of genes for essential host processes and antiviral responses. During viral infection, the host cell responds by halting nonessential transcription while upregulating defense genes. For example, upon detection of an invading virus through pathogen associated molecular patterns (PAMPs), the host cell drives interferon transcription, which in turn initiates a cascade of interferon-stimulated gene (ISG) expression to defend the infected organism (55, 56). The DNA sensing ISG known as IFI16 causes H3K9me3 to increase specifically on the viral genome at lytic gene promoters in an ICP0-dependent manner (57, 58). This interesting finding indicates that IFI16 promotes deposition of silencing marks on the incoming viral genome to prevent transcription of viral genes. While interferon responses are silenced, lytic HSV-1 DNA is marked with H3K4me3 and H3K9Ac, consistent with active viral transcription (59, 60). This highlights the complexity of the post-translation histone modifications in proviral and antiviral transcription.

During lytic infection, CTCF is recruited to viral replication compartments and is required for HSV-1 transcription (61). It is thought that CTCF arranges chromatin in a manner that brings enhancers physically nearer to lytic viral genes, thus driving transcription of essential viral genes. Indeed, knockdown of cohesin complex components causes reduced lytic gene transcription and lower recruitment of RNA polymerase II (RNA Pol II) to viral lytic genes (62). Thus, reduced RNA Pol II recruitment could be a result of more physical distance between enhancers and viral transcription start sites. These findings support a model in which CTCF and cohesin-mediated chromatin folding activate viral lytic genes.

**H3K4me3:**

denotes the post-translational modification of trimethylation on histone H3 at lysine 4 and is generally associated with transcriptional activation

**H3K9Ac:**

denotes the post-translational modification of acetylation on histone H3 at lysine 9 and is generally associated with transcriptional activation

#### 2.1.2. Chromatin dynamics during herpes simplex virus latency and reactivation.

During latency, heterochromatin marks associate with HSV-1 genomes (63, 64). The polycomb repressive complex 2 (PRC2) and H3K27me3 begin associating with the HSV-1 genome in vivo several days after latency is established (65). In fact, inhibition of the H3K9me3 demethylase or H3K27me3 demethylase reduces reactivation in mouse models because reducing the removal of the corresponding silencing mark promotes the repressive state of the latent HSV-1 genome (66, 67). EZH2 or enhancer of zeste homolog 2 is the PRC2 subunit that deposits the H3K27me3 mark (68). Arbuckle et al. (69) found that EZH2 inhibition induces expression of a set of ISGs that in turn reduce HSV-1 transcription while also promoting host defenses. Thus, small molecule inhibitors to epigenetic modifications offer a promising avenue for treatment to prevent HSV reactivation.

Latent HSV also takes advantage of CTCF function. During latent infection, CTCF associates with the latency-associated transcript (LAT), whose tightly controlled transcription regulates reactivation. Remarkably, there are seven CCCTC sites on the LAT gene. Upon deletion of one CCCTC site, LAT becomes increasingly associated with repressive histone marks (70). While a more repressive chromatin state would be predicted to produce lower LAT levels and promote reactivation, mutation of this specific CTCF binding site on the LAT gene resulted in less reactivation. Conversely, depletion of other sites often resulted in more reactivation (71–73). Thus, the outcome of loss of CTCF binding is likely dependent on which specific site is affected. These findings indicate that the interaction of viral genomes with host factors such as CTCF during latency and reactivation has many facets that are yet to be deciphered.

### 2.2. Nucleolar Disruption During Herpes Simplex Virus Infection

Early in HSV-1 infection the nucleolus becomes more electron dense, appearing compact before fragmenting later during viral replication (36). Nucleolar proteins including nucleolin, fibrillarin, B23, and upstream binding factor (UBF) become diffuse throughout the nucleus during infection (74–76). The redistribution of nucleolar proteins B23 and nucleolin is dependent on the HSV protein UL24 (75, 76). Loss of UL24 or knockdown of nucleolin results in reduced titers and increased nuclear capsids, suggesting the inability to disrupt the nucleolus may be detrimental to egress. Viral proteins VP22 and Us11 also interact with nucleolin (77, 78), but their role in nucleolar rearrangement is less clear. As study of the nucleolus progresses, it will be interesting to follow how virus infection affects nucleolar biology and how nucleolar discoveries may indicate areas of viral vulnerabilities.

## 3. CYTOMEGALOVIRUS REARRANGEMENT OF THE NUCLEAR COMPARTMENT

Cytomegalovirus (CMV) is a betaherpesvirus defined by a slow lytic replication cycle. CMV infects a majority of the world’s population with seropositivity reaching greater than 90% depending on the country of study (79). Many CMV infections are asymptomatic or result only in minor cold-like symptoms (80). Perhaps due to this low severity of illness, CMV is less well-known than other herpesviruses in the general population. Nevertheless, CMV is one of the leading infectious causes of birth defects with congenital CMV occurring in an estimated 1 in 200 births. As many as 20% of these congenital infections result in disabilities, most commonly deafness (81). The latent phase of CMV is established in monocyte precursors, and CMV reactivation can cause severe disease in immunocompromised individuals. CMV infection is a major source of complications following bone marrow or solid organ transplant (82) and a common culprit of morbidity in AIDS patients (83). In these cases of severe disease, treatment options are limited (84).

CMV infection causes large-scale cellular rearrangement during lytic infection. This rearrangement includes reorganization of the host Golgi and endosomes to form a cytoplasmic viral-induced assembly compartment (vIAC) where virions undergo maturation (85). At the same time the vIAC is formed, the host nucleus acquires a distinct kidney bean shape that wraps around the vIAC, with host chromatin polarized toward the vIAC (86) (Figure 1). These cellular changes are critical for progeny production because high levels of infectious CMV progeny cannot be produced without nuclear remodeling and the formation of a compact, spherical vIAC (87, 88). Interestingly, expression of CMV NEC proteins UL50 and UL53 is sufficient to initiate remodeling of host nuclei (89). Thus, nuclear structure is intimately tied to viral productivity, and research in this area is likely to uncover important results relevant for multiple viruses.

**Viral-induced assembly compartment:**

CMV-induced cytoplasmic structure composed of endoplasmic reticulum, Golgi, and endosomes where CMV progeny mature

### 3.1. Reorganization of Host Chromatin During Cytomegalovirus Infection

One of the first proteins expressed by CMV is immediate-early protein 1 (IE1), a transcriptional activator that promotes viral gene expression and replication (90). IE1 directly interacts with host core histones through preferential binding of the acidic patch on the H2A-H2B region (90). This interaction causes disruption of host chromatin architecture and was suggested to impair higher-order compaction of the host genome (91). Furthermore, this interaction may serve to promote inheritance of latent CMV genomes through cell divisions via tethering (92). These results suggest that IE1 evolved to harness host chromatin structure to promote infection, though the direct benefit to viral progeny production remains unclear.

On a global scale, it is becoming increasingly clear that CMV infection causes specific reorganization of host chromatin. Procter et al. (86) and others showed that chromatin associated with heterochromatin markers H3K9me2 and H3K9me3 are polarized toward the vIAC and excluded from the viral replication compartment (93). In contrast, chromatin associated with active marker H3K4me3 and heterochromatin marker H3K27me3 are not polarized and remain diffuse in the nucleus (86). In addition to affecting histone mark arrangement within the nucleus, CMV infection causes reduction in transcription and messenger RNA (mRNA) processing of core histones (94). Together these studies suggest that control of histone levels and their modifications may be a critical aspect of CMV infection that has yet to be fully revealed.

### 3.2. Chromatin Factors on the Cytomegalovirus Genome During Lytic and Latent Infection

During lytic replication, CMV genomes are marked with both active and repressive histone modifications at different stages of infection (93, 95–97). These stages are sequentially separated into expression of immediate-early (IE), early (E), and late (L) gene transcripts. During the first phase of infection and transcription of IE genes, repressive marks are associated with genes that are expressed later, namely E and L gene promoters (98, 99). Thus, premature expression of E and L genes is avoided by utilizing the host repressive machinery associated with marks like H3K27me3 (96, 97). As IE gene transcription begins, active marks H3K9Ac and H3K14Ac are deposited on the major immediate-early promoter (MIEP). Further, IE1 prolongs viral transcription by inhibiting cellular deacetylase activity at active viral promotors (100). Following viral replication, additional active marks are deposited on CMV E and L gene promoters while repressive marks at these promoters are reduced (95), promoting a shift in transcription toward a later stage of infection. Interestingly, the MIEP includes a CTCF binding site that upon deletion enhances IE transcript levels, indicating that CTCF acts as an insulator in this setting (101). Further, depletion of CTCF results in a 50-fold increase in progeny production during lytic infection (101), highlighting the importance of this factor during CMV infection.

The CTCF binding site in the MIEP of CMV is also important during latency as CTCF binds in the MIEP to repress IE1 and IE2. Furthermore, viral encoded protein Us28 induces increased expression of CTCF during latent infection, likely to help maintain latency (102). EZH2 inhibition results in strong reactivation of CMV, indicating that H3K27me3 on the viral genome also supports latency (103). These findings underscore that CMV latency is dependent on host factors actively maintaining heterochromatin.

### 3.3. Nucleolar Dynamics During Cytomegalovirus Infection

In CMV-infected cells, dense fibrous structures that resemble the nucleolus persist through viral replication and can outline the regions producing viral capsids (104, 105). Despite the apparent maintenance of nucleolar structure during CMV infection, core nucleolar proteins become diffuse throughout the nucleus beginning early in infection. Late gene expression results in mislocalization of nucleolin, which is required for targeting viral UL44 to sites of viral replication to promote DNA synthesis (106). Loss of nucleolin results in reduced viral titers, suggesting that either nucleolin or the nucleolus is important for CMV replication. The viral protein UL31 (not homologous with HSV UL31 discussed earlier) may play a key role in this nucleolar reorganization by promoting mislocalization of nucleolin and UBF (107). Thus, it is clear that several nucleolar proteins are important for viral replication even though further study is needed to fully understand the role of nucleolar structure during CMV infection.

## 4. ADENOVIRUS INFECTION IN THE NUCLEUS

Adenoviruses are small double-stranded DNA viruses that cause multiple diseases, including gastroenteritis, conjunctivitis, and respiratory infections (31). While generally viewed to cause a self-limiting infection, adenoviruses are particularly harmful for immunocompromised individuals, especially those undergoing stem cell therapy and organ transplants (108, 109). Worryingly, in the past few years, there have been several reported cases of acute hepatitis in children, most of whom tested positive for adenovirus infection (110, 111). While the exact cause of hepatitis cannot be definitively attributed to adenovirus, this trend shows that there is much we have yet to understand about how adenovirus causes disease. Though there is an adenovirus vaccine, it is not commonly distributed outside the US military (112), and adenovirus treatments are limited (113).

**Figure 2:**
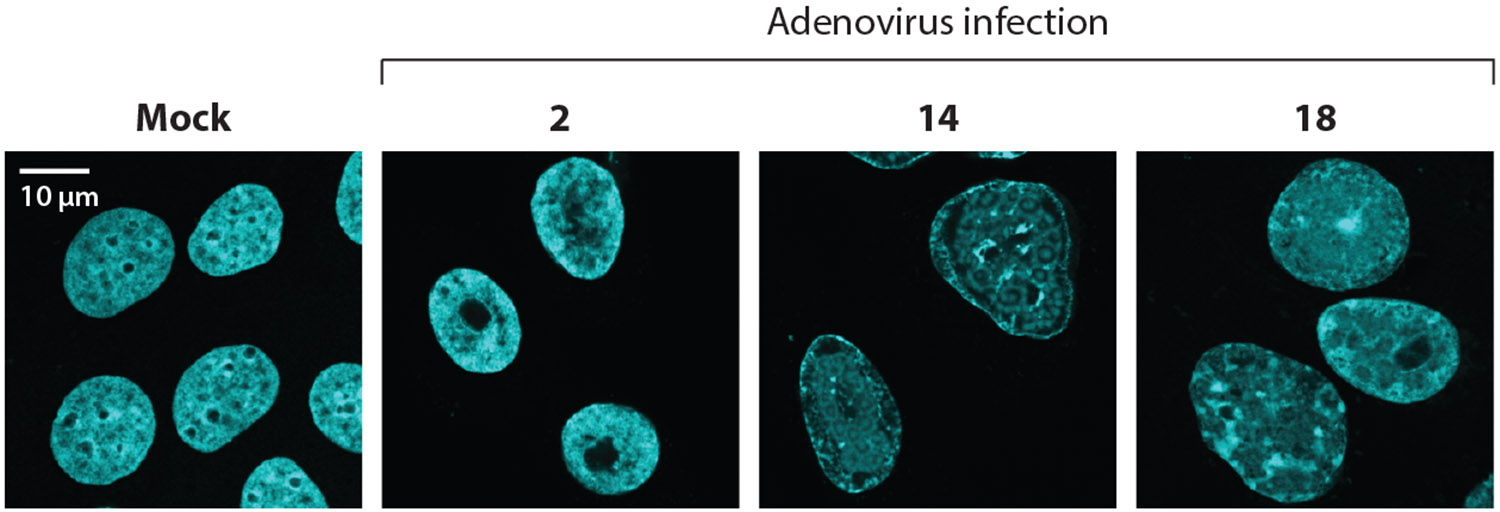
Adenovirus infection causes host chromatin reorganization during infection. Immunofluorescence images show adenovirus type 5 infection in A549 lung epithelial cells with changes to DAPI appearance (*cyan*) over the course of infection. Time points in hours post infection are as indicated. Abbreviation: DAPI, 4′,6-diamidino-2-phenylindole.

At the structural level of the nucleus, adenovirus infection induces several changes. Adenovirus infection causes a general enlargement of the nucleus, which has been reported in many cases and is easily visualized by DAPI (4′,6-diamidino-2-phenylindole) staining in immunofluorescence microscopy (114, 115) (Figure 2). Adenovirus infection takes over the nucleus and forms replication centers that are sites of genome accumulation, transcription, and RNA splicing and are near sites of newly formed virions (116). Replication centers begin as several small foci throughout the nucleus, visualized by staining of the viral single-stranded DNA binding protein, DBP (116). As infection progresses, these replication centers grow and form larger ring-like centers. At late stages of infection, a large late viral accumulation center (LVAC) marked by protein V forms and occupies most of the center of the nucleus (117). Recently, Pfitzner et al. (117) showed using transmission EM and fluorescence recovery after photobleaching that the LVAC is full of a para-crystalline array of virions in a rigid, immobile structure. These replication compartments together with the LVAC occupy most of the nucleus, likely providing sufficient space for the production of thousands of progeny virions.

### 4.1. Adenovirus Remodeling of Host Chromatin

At the chromatin level, adenoviruses express multiple proteins that change the chromatin landscape of the host cell. Most notable of these proteins are E1A and protein VII. The first adenovirus protein expressed during infection, E1A, interacts with multiple host proteins, such as the histone acetyltransferases p300 and CREB-binding protein (CBP) (118). The interaction of E1A with p300 and CBP causes a global decrease in H3K18ac on the host genome, promoting transition into a viral-induced S-like phase (119–121). E1A localizes to several host promoters and causes transcriptional changes including increased expression of cell cycle–regulated genes, inhibition of immune-related genes, and decreased expression of genes associated with differentiation and development (122–124). These changes exemplify the role of E1A as a transformative agent that optimizes the cell for viral replication.

As one of the viral core proteins, protein VII is delivered with the viral genome to the nucleus and is subsequently expressed at high levels during late stages of infection. Protein VII delivered with the genome interacts with and recruits E1A to the viral genome to initiate transcription of early genes (125). Protein VII is a part of the major late transcription unit of the adenoviral genome and localizes to and distorts host chromatin late during infection (114). In addition, protein VII interacts with numerous host chromatin-associated proteins, such as high mobility group box 1 (HMGB1) (114) and host histone chaperone SET (126, 127). HMGB1 is a nuclear cotranscription factor and extracellular alarmin protein (128). Protein VII sequesters HMGB1 in the host chromatin and through this interaction selectively dampens interferon responses (129). Host protein SET, also known as TAF1β, facilitates deposition of histones onto the viral genome during early stages of infection (130). This protein VII-SET interaction promotes the delay of cell cycle progression to maintain the viral S phase and dampen the DNA damage response (DDR) to infection (114, 115). Thus, adenovirus protein VII utilizes multiple strategies through host chromatin to dampen the host immune response and promote viral proliferation.

Another early viral protein, E4orf3, interacts with promyelocytic leukemia nuclear bodies (PML bodies), changing their structure from ring shaped to tracks (131). Several proteins with innate immune functions are normally found associated with PML bodies, including the PML protein, which itself is an ISG. Knockdown studies of PML and/or Daxx, another ISG that associates with PML bodies, rescued replication of an E4orf3 mutant adenovirus (132). Furthermore, DDR proteins of the MRN complex are also relocalized by E4orf3. In uninfected cells, these proteins are found dispersed throughout the nucleus, which will facilitate the concatamerization of adenovirus genomes. However, E4orf3 relocalizes these proteins to distinct foci separated from sites of adenovirus genome replication, preventing them from inhibiting replication (133). Lastly, E4orf3 interacts with the N terminus of E1A, which enhances E1A promoter occupancy at viral genes and increases expression of the viral early gene E1, E2, and E3 (134). While E4orf3 is not known to directly interact with host chromatin, the structural reorganization of nuclear components is critical for the success of adenovirus infection.

### 4.2. Nucleolar Dynamics During Adenovirus Infection

Adenovirus infection disrupts the nucleolus, and many nucleolar resident proteins are relocalized to VRCs such as NPM1/B23, UBF, nucleolin, and Nopp140 (135–140). While many nucleolar proteins remain to be investigated in the context of adenovirus infection, NPM1 has been the focus of multiple studies (139–141). NPM1 is a multifunctional protein involved in ribosome biogenesis, mRNA processing, chromosome remodeling, and DNA repair pathways (142). During adenovirus infection the core proteins protein V and VII directly interact with NPM1, where NPM1 acts as a histone chaperone with protein VII, helping assemble chromatin on viral DNA (141). NPM1 also likely functions in virion assembly as it was found by immunoelectron microscopy to localize near empty capsids (139). Knockdown of NPM1 inhibits viral infection, but downstream of genome replication (140), further supporting its potential function in virion assembly. Together, these studies demonstrate the importance of nucleolar host proteins in adenovirus infection.

### 4.3. Nuclear Lamina Changes During Adenovirus Infection

Adenovirus infection causes the nuclear lamina to destabilize and break down, resulting in ruptures to the nuclear membrane (143). The viral E3 ORF encodes the adenovirus death protein (ADP), which is involved in the eventual lysis of the host cell membranes to release progeny virions (144). Interestingly, Pfitzer et al. (143) showed that adenovirus type 5 with or without ADP resulted in similar breakdown of the nuclear lamina and ruptures in the nuclear membrane, suggesting other factors are at play. How adenovirus causes this disruption to escape the nucleus remains to be determined.

## 5. DISCUSSION AND OUTLOOK

**Figure 3:**
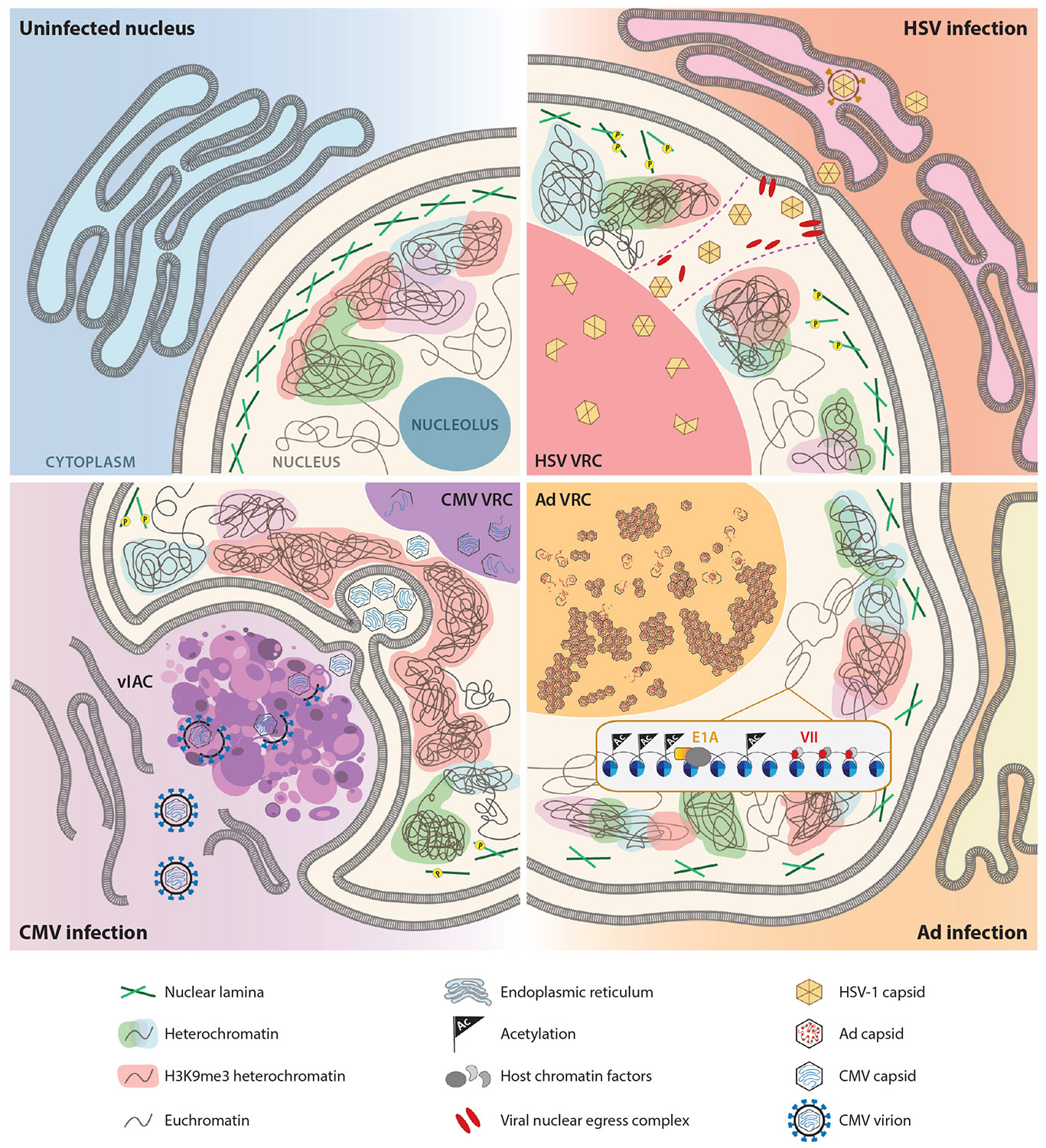
Schematic of the large-scale disruptions discussed in this review that occur during HSV, adenovirus, and CMV infections. In uninfected cells, heterochromatin predominantly resides along the nuclear periphery and nucleolus. The nucleolus is a phase-separated compartment where ribosomes are assembled. Nuclear lamina and peripheral heterochromatin support the nuclear envelope. In these virus infections, the nuclear volume expands and host chromatin structure is reorganized as large viral replication centers become the dominant nuclear feature. In herpes simplex virus infection, chromatin is redistributed and condensed at the periphery as new regions of heterochromatin are formed. Viral capsids pass through chromatin channels to the nuclear membrane for egress. During adenovirus infection, viral proteins E1A and protein VII remodel host chromatin and the replication center takes over the nuclear compartment to generate viral progeny, which are released in cellular lysis. In cytomegalovirus infection, the nucleus forms a distinct kidney bean shape and H3K9me3-associated heterochromatin is polarized toward the cytoplasmic viral-induced assembly compartment, where egressing virions mature. Structures depicted are described in the key. Abbreviations: Ad, adenovirus; CMV, cytomegalovirus; HSV, herpes simplex virus; vIAC, viral-induced assembly compartment; VRC, viral replication center.

Nuclear replicating viruses perturb both genomic and nuclear architecture to generate viral progeny (Figure 3). Advances in techniques for sequencing and imaging have significantly advanced our understanding of virus infection in the nucleus (summarized in Table 1). The literature discussed here highlights the complex dual role of chromatin as the transcriptional source of the resident genome and a physical structure in the nucleus during viral infection. The next steps in the field utilizing recent gene-editing advances together with increasingly sophisticated bioinformatics pipelines will allow for tracking viral-induced changes to chromatin structure and transcription throughout infection. Particularly, it is the combination of multiple techniques that will allow for a holistic understanding of the effect viral infection has on the nucleus, and conversely, how chromatin mechanisms have evolved to combat virus infection.

Although we have focused on herpesviruses and adenoviruses in this review, similar techniques have revealed how viruses generally manipulate host chromatin and biology. For example, studies using Hi-C and super-resolution microscopy identified that hepatitis B virus closely associates with H3K4me3-rich regions of the host genome (145, 146). Further, mass spectrometry of papillomaviruses revealed that histones packaged inside virions with the viral genome are predominantly associated with activating histone marks, suggesting that incoming viral genomes are poised for gene expression (147). Given the importance of chromatin in all aspects of cellular function, it is unsurprising that cytoplasmic RNA viruses such as severe acute respiratory syndrome coronavirus 2 also disrupt host chromatin architecture to evade immune responses (148). Thus, the discoveries laid out here, and those that will undoubtedly surface as technology advances, have enormous potential to pinpoint host vulnerabilities and viral immune evasion strategies.

## Figures and Tables

**Table 1:** Description of current technologies used to study how viruses manipulate host chromatin and nuclear architecture

Technique	Purpose	Comments	Example(s)
Genomic techniques			
**Chromatin immunoprecipitation (ChIP)**	To investigate localization of proteins on the genome	Primarily used to map transcription factors and histone modifications Requires a specific antibody and is challenging to normalize replicating viral genomes High numbers of cells required	65, 149
**Cleavage Under Targets and Tagmentation (CUT&Tag)/CUT&Run**	To investigate localization of proteins on the genome with higher resolution and lower background compared to ChIP	Uses intact nuclei to reflect the native chromatin state more accurately Requires fewer cells than ChIP but still relies on antibody specificity	44, 150
**Chromosome confirmation capture (3C)/4C**	To examine the 3D structure of chromatin at a specific region	Can be used to identify physical interactions between viral and host chromatin Requires advanced bioinformatics and large amounts of cells	151, 152
**Hi-C**	To investigate all the chromatin interactions in a cell in an unbiased manner	Similar to 3C/4C in that it requires advanced bioinformatics and large amounts of cells Can be costly to sequence at the depth required to make biological conclusions	146
Imaging techniques			
**Confocal microscopy/live imaging**	To investigate subnuclear localization of host and viral proteins and visualize how they change during infection	Z stacks can be acquired for 3D rendering Relies on specific antibodies that can have cross-reactivity with viral proteins	40, 45, 46, 114
**Super-resolution microscopy**	To visualize intermolecular interactions with resolution beyond the diffraction barrier	Requires specialized equipment and extensive protocols	145
**Electron microscopy**	To visualize cellular organelles and structures using an electron beam	Micrographs of densities have high resolution and can detect individual capsids and heterochromatin Requires specialized equipment, and 3D rendering is challenging	44, 153
Combination/other techniques			
**Mass spectrometry**	To identify proteins and their post-translational modifications using the mass-to-charge ratio	Data sets obtained from isolated proteomes or proteomic analysis can be very powerful to define changes to host cells during infection Requires specialized equipment and analysis	154, 155
**Isolation of proteins on nascent DNA (iPOND)**	To determine the proteins associated with replicating DNA genomes via 5-ethynyl-2′-deoxyuridine incorporation	Can be used to identify proteins interacting with viral genomes during replication Requires in-depth follow-up via western blotting or mass spectrometry Requires large amounts of cells	137, 156
**Atomic force microscopy**	To determine physical properties of a sample using a probe to apply force	Can be used to determine changes to nuclei upon infection or virion stability Requires specialized equipment and analysis	43

## SUMMARY POINTS

1. Nuclear and chromatin structures are tightly regulated features of healthy cells that control gene expression and support nuclear integrity.
2. Post-translational modifications, or marks, on histone proteins are key for host and viral gene expression regulation.
3. Nuclear proteins, including nucleolar proteins and structural factors such as CTCF, are critical for productive viral infections.
4. Nuclear replicating DNA viruses, such as adenoviruses and herpesviruses, cause large-scale disruptions to the nucleus and host chromatin that promote viral proliferation.
5. During lytic replication, herpesvirus genomes are peppered with histone marks associated with active transcription while repressive histone marks help establish and maintain latency.
6. Adenoviruses hijack host nuclear factors to alter the host epigenetic landscape and disrupt nuclear substructures to promote infection.
7. Cytoplasmic replicating viruses also disrupt host chromatin architecture.
8. Technological advances continue to allow deeper investigation into viral hijacking of host chromatin factors.

## FUTURE ISSUES

1. What are the mechanisms by which host chromatin responds to the stimulus of infection?
2. How do nuclear viruses exploit stress-induced host chromatin changes for viral benefit?
3. How do other nuclear replicating viruses contend with host chromatin as a barrier to egress?
4. How do viruses that maintain latency navigate the balance of CTCF’s activating and insulating functions?
5. Why are some chromatin marks strongly polarized during cytomegalovirus infection while others are not? Which host genes are localized toward the viral-induced assembly compartment, and how does this affect expression?
6. What is the function of nucleolar factors during nuclear virus infection?
7. How can we leverage viral mechanisms of chromatin manipulation to develop targeted therapeutics for host and viral diseases?

## ABBREVIATIONS LIST

ADP: adenovirus death protein
CBP: CREB-binding protein
CMV: cytomegalovirus
CTCF: CCCTC-binding factor
EM: electron microscopy
EZH2: enhancer of zeste homolog 2
FIREs: frequently interacting regions of chromatin
HMGB1: high mobility group box 1
HSV: herpes simplex virus
IE1: immediate-early protein 1 in CMV
INM: inner nuclear membrane
ISG: interferon-stimulated gene
LAT: latency-associated transcript in HSV
LINC: linker of nucleoskeleton and cytoskeleton
LVAC: late viral accumulation center in adenoviruses
MIEP: major immediate-early promotor in CMV
NEC: nuclear egress complex
PAMP: pathogen associated molecular pattern
PML bodies: promyelocytic leukemia nuclear bodies
PRC2: polycomb repressive complex 2
rRNA: ribosomal RNA
TAD: topologically associating domain
UBF: upstream binding factor
vIAC: viral-induced assembly compartment in CMV
VRC: viral replication center
